# Determination of Spearman Correlation Coefficient (*r*) to Evaluate the Linear Association of Dermal Collagen and Elastic Fibers in the Perspectives of Skin Injury

**DOI:** 10.1155/2018/4512840

**Published:** 2018-05-02

**Authors:** Naveen Kumar, Pramod Kumar, Satheesha Nayak Badagabettu, Melissa Glenda Lewis, Murali Adiga, Ashwini Aithal Padur

**Affiliations:** ^1^Department of Anatomy, Melaka Manipal Medical College, Manipal Academy of Higher Education, Manipal Campus, Manipal 576104, India; ^2^King Fahad Central Hospital, Jazan 82666, Saudi Arabia; ^3^Indian Institute of Public Health Hyderabad (IIPHH), Madhapur, Hyderabad 500033, India; ^4^Department of Physiology, Kasturba Medical College, Manipal, Manipal Academy of Higher Education, Manipal 576104, India

## Abstract

**Background:**

Difference in scar formation at different sites, in different directions at the same site, but with changes in the elasticity of skin with age, sex, and race or in some pathological conditions, is well known to clinicians. The inappropriate collagen syntheses and delayed or lack of epithelialization are known to induce scar formation with negligible elasticity at the site of damage. Changes in the elasticity of scars may be due to an unequal distribution of dermal collagen (C) and elastic (E) fibers.

**Materials and Methods:**

Spearman correlation coefficients (*r*) of collagen and elastic fibers in horizontal (H) and in vertical (V) directions (variables CV, CH, EV, and EH) were measured from the respective quantitative fraction data in 320 skin samples from 32 human cadavers collected at five selected sites over extremities.

**Results:**

Spearman's correlation analysis revealed the statistically significant (*p* < 0.01) strong positive correlation between C_H_ and C_V_ in all the areas, that is, shoulder joint area (*r* = 0.66), wrist (*r* = 0.75), forearm (*r* = 0.75), and thigh (*r* = 0.80), except at the ankle (*r* = 0.26, *p* = 0.14) region. Similarly, positive correlation between E_H_ and E_V_ has been observed at the forearm (*r* = 0.65, moderate) and thigh (*r* = 0.42, low) regions. However, a significant moderate negative correlation was observed between C_V_ and E_V_ at the forearm (*r* = −0.51) and between C_H_ and E_H_ at the thigh region (*r* = −0.65).

**Conclusion:**

Significant differences of correlations of collagen and elastic fibers in different directions from different areas of extremities were noted. This may be one of the possible anatomical reasons of scar behavior in different areas and different directions of the same area.

## 1. Introduction

Collagen and elastic fiber contents of the dermis perform a complimentary role in maintaining skin shape and firmness. While rigidity of the skin is provided by the collagen content, the elastic fiber content maintains the skin elasticity by forming a three-dimensional network between the collagen fibers. Injury to the skin that results in damage to the collagen network will often have an adverse effect on wound healing. Therefore, the mechanical quality of the skin is mostly achieved by collagen fibers with their strong tensile strength and cross-link appearance [1]. Elastin, though remarkably less than collagen, plays a critical role in wound healing mechanism as evidenced by the following observations by clinicians:

- Transplantation of the epidermis and superficial portion of the dermis from healthy skin (split skin graft) is the most successful and standard treatment in the case of full-thickness loss of the skin. The major drawback of this approach is that the healed split skin graft usually lacks elasticity and increased fragility associated with sensory disturbances. These changes in the transplanted split skin graft may be attributed to changes in the arrangement and content (ratio) of collagen and elastic fibers together with the extracellular matrix proteins [2].
- Langer's line or skin cleavage line has been a well-known direction for making incisions to obtain an esthetic result for many years. Besides, there are many other concepts of lines on the skin that have been demonstrated and made the idea of Langer's line debatable. According to Albert Borges, this is the single best choice of line; however, it is still questionable to fulfil complete satisfaction of wound healing [3]. Nevertheless, the concept of Langer's line over the pattern of arrangement of dermal collagen in a particular direction cannot be ruled out.
- The varied quantity and asymmetric distribution of collagen and elastic fibers in different orientations of the skin plane are known to manipulate the scar appearance [4–6].

In addition to the above observations, it is presumed that relative correlations between these two predominant connective tissue fibers could also be equally responsible for the unpredictable behavior of a scar, despite the incisions made in accordance with the standard lines of choice. Hence, the present work has been undertaken to determine the role of dermal collagen-elastic correlation in the region of upper and lower extremities of the human body. The descriptions presented herewith serve as an essential tool in the esthetic therapy as well as in scar management.

## 2. Materials and Methods

Skin excision tissues were collected from five different areas involving both extremities from formalin embalmed adult male human cadavers with the age range between 55 and 70 years. The samples from each region were obtained in two directions, namely, horizontal and vertical. Institutional human ethical clearance was obtained through the proper standard protocol before the study.

### 2.1. Topographic Sites of Sample Collection

*Shoulder Area*. At the region just lateral to the surface projection overlying the acromion process of the scapula, skin tissue that was taken along the circumferential line of joint was considered as “horizontal,” while that perpendicular to it was considered as “vertical.”

*Wrist*. Over the wrist area, skin samples in two directions were taken at the site of proximal crease line of the flexor surface. 

*Ankle*. Skin tissues were obtained at the site about one centimeter above the insertion of the tendo calcaneus.

*Forearm*. This is located along the midline on the flexor compartment of the forearm between the midpoints of the elbow joint and the wrist joint.

*Thigh*. This is collected at the midpoint of the thigh between the pubic tubercle and the medial tibial condyle.

### 2.2. Histopathological Processing

The skin samples were processed for the special Verhoeff–van Gieson (VVG) stain. VVG is employed for the selective demonstration of elastic fibers that appear black in the background of the pink collagen fiber content.

### 2.3. Image Analysis Details

The digital images of VVG stained sections were acquired at 20x magnification using ProgRes CapturePro 2.1 Jenoptik microscopic camera with the resolution of 694 × 516 VGA. The collagen fiber tissue which is expressed in pink shades in the image was segmented out by appropriately adjusting the color settings in the software. A similar configuration was employed for the study of all the photos. The area occupied is calculated by the software regarding the number of pixels. In the same way, the elastic fibers which take up black shades upon staining were also selectively segmented, and the area occupied by them was also calculated in terms of the number of pixels. These values were further calculated based on the percentage area occupied by these fibers that is referred to as quantitative fraction.

The images were analyzed using the software named “TissueQuant,” which is designed for color quantification in the image [7, 8]. This software provides the facility to choose a color for selectively measuring the areas in the image.

### 2.4. Spearman Correlation Analysis

Spearman correlation coefficient (*r*) was estimated to determine the linear association between the following variables:

- C_H_ and C_V_ (collagen content between horizontal and vertical directions).
- C_H_ and E_H_ (between collagen and elastic fiber contents in the horizontal direction).
- C_V_ and E_V_ (between collagen and elastic fiber contents in the vertical direction).
- E_H_ and E_V_ (elastic fiber content between horizontal and vertical directions).

The outcome results were interpreted according to the degree of association as strong (*r* = 0.7–1), moderate (*r* = 0.5–0.7), or low (*r* = 0.3–0.5) after taking significant correlation (*p* < 0.01 or *p* < 0.05) values into consideration.

## 3. Results

**Figure 1:**
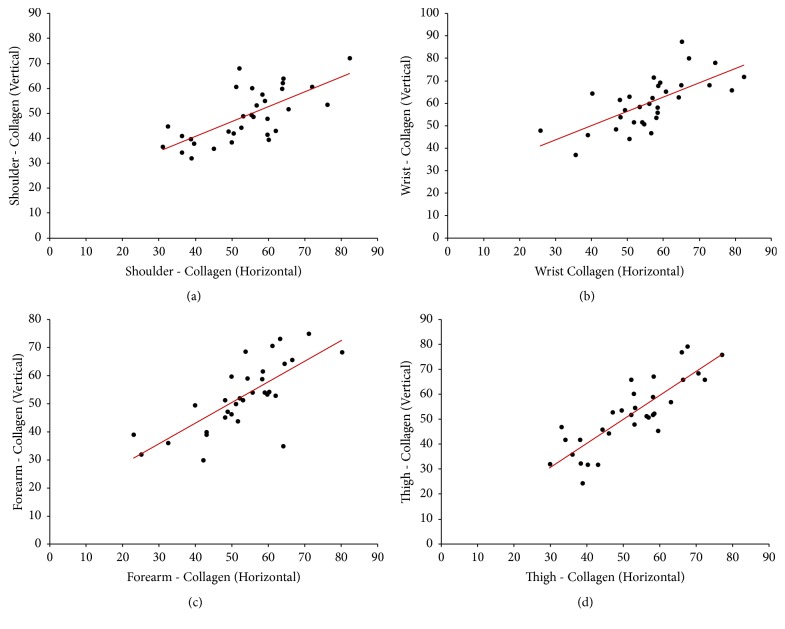
Scatter plot showing significant (two-tailed) Spearman positive correlation of collagen in horizontal versus vertical directions at (a) shoulder joint area (moderate; *r* = 0.66), (b) wrist (strong; *r* = 0.75), (c) forearm (strong; *r* = 0.75), and (d) thigh (strong; *r* = 0.80) areas.

**Figure 2:**
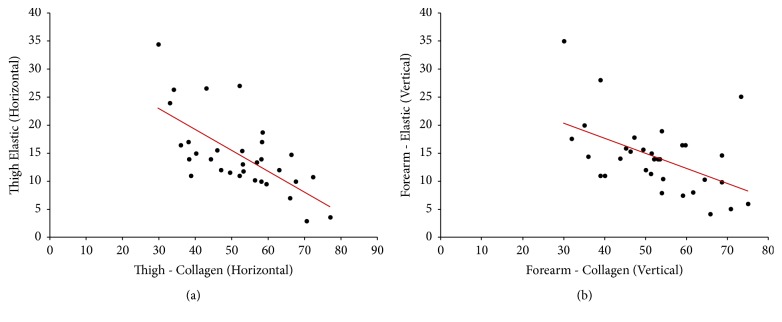
Scatter plot showing significant (two-tailed) Spearman negative correlation between collagen and elastic fibers in horizontal direction in the (a) thigh (moderate; *r* = −0.65) and negative correlation between collagen and elastic fibers in the vertical direction at the (b) forearm (moderate; *r* = −0.51).

**Figure 3:**
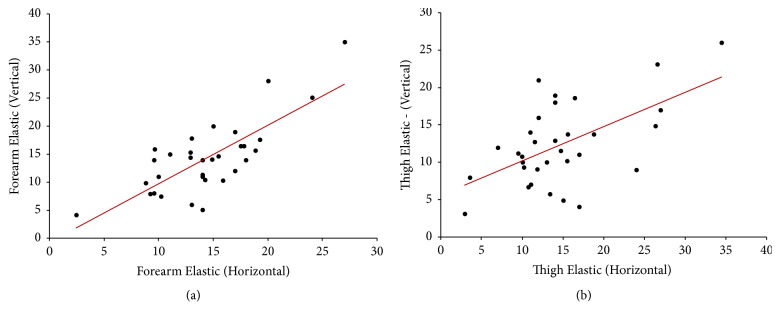
Scatter plot showing significant (two-tailed) Spearman positive correlation of elastic fibers between horizontal and vertical directions at (a) forearm (moderate; *r* = 0.65) and (b) thigh (low; *r* = 0.42) areas.

Descriptive statistics of Spearman's correlation coefficient (*r*) and the level of significance (*p*) as tested at the extremity region were tabulated (Table 1) and the comprehended correlation data between the significant variables were depicted (Table 2). Significant correlations were represented as scatter plots (Figures 1–3).

Spearman's correlation analysis revealed the statistically significant strong positive correlation between C_H_ and C_V_ in all the areas under study [shoulder joint (*r* = 0.66, *p* < 0.01), wrist (*r* = 0.75, *p* < 0.01), forearm (*r* = 0.75, *p* < 0.01), and thigh (*r* = 0.80, *p* < 0.01)] except at the ankle (*r* = 0.26, *p* = 0.14).

Moderate negative correlation between C_H_ and E_H_ was at the thigh area (*r* = −0.65, *p* < 0.01) and between C_V_ and E_V_ at the forearm (*r* = −0.51, *p* < 0.05) areas, which were found to be significant.

Significant positive correlation was also observed between E_H_ and E_V_ at forearm (*r* = 0.65, *p* < 0.01, with moderate correlation) and thigh (*r* = 0.42, *p* < 0.05, with low correlation) areas of extremities.

## 4. Discussion

A study has shown that, in the process of skin graft, an imbalance between collagen synthesis and its cross-linkage and/or the lack of dermal appendages make it relatively less elastic and vulnerable to immobility (contracture) [2]. The progression of healing following skin grafting/skin injury is similar [9].

Among the joint areas, positive correlations between C_H_ and C_V_ were observed at shoulder joint (moderate) and wrist areas (strong). No other significant correlation pattern was found. The ankle did not show any significant correlations among the variables tested. On the other hand, both the forearm and thigh areas did show a strong positive correlation between C_H_ and C_V_ and positive correlations (medium positive for forearm and low positive for thigh) between E_H_ and E_V_. This observation could be due to strain and frequency of movement of joint.

Further, forearm exhibited a moderate negative correlation between C_V_ and E_V_ whereas thigh area showed a moderate negative correlation between C_H_ and E_H_. Thus, collagen and elastic fiber content will have a negative correlation with each other in the horizontal direction of thigh area, whereas a similar relation between them could be seen in the vertical direction at forearm. This difference of trend could be due to the difference of direction of strain due to supination and pronation specific to the forearm and flexion and extension at the knee.

The results of quantitative fraction analysis in these sites exhibited significantly higher content of elastic fibers in the vertical direction (E_V_) at shoulder and wrist areas. But for the collagen content, there was a higher proportion in horizontal (C_H_) direction at shoulder joint area and in vertical (C_V_) direction for wrist area (Table 3). The maximum effect of burst force and minimum effect of stretch force at the shoulder joint and vice versa at the wrist are said to be responsible for the alternative dominance of collagen content, but the uniform predominance of elastic fiber content was ruled out.

The elastic fiber content of the thigh is higher in horizontal (E_H_) direction, and it is higher in the vertical direction (E_V_) at the ankle area. Both areas, however, have shown no difference or a negligible difference for collagen content in either of the courses (C_H_ and C_V_) specified. The stretch force exerted by slow circumferential expansion of the thigh due to changes in the bulk of thigh muscles during knee movements and compensatory effect of elastic fiber on excess stretching and laxity at ankle area during plantar flexion/dorsiflexion indicated the alternative predominant pattern of elastic fiber content [6].

Various clinical studies also support our assumption that relative correlation between the connective tissue fibers, apart from other reasons, could also be responsible for the unpredictable behavior of a scar.

An interventional study performed on excision wounds of the trunk and extremities when treated with bilayered closure procedure involving the deep dermis (that avoids stretching of scar) revealed an overall better appearance of the scar than subcuticular epidermal closure procedure of the same [10].

A hypertrophic scar is familiar in knees and ankles due to the constant tension (stretching during more frequent movements during locomotion) [11, 12], whereas the shoulder is said to be a common site for keloid [13]. It could be attributed to the dominance of both Cv and Ev at the shoulder but for Ev at the ankle only (Table 3). A study on evaluating the pattern of skin cleavage lines of cadaveric feet suggested that incision should be along or parallel to skin cleavage lines. If this is not entirely possible due to operative requirements, to get an optimal result as far as the scar is concerned, at least a large part of the incision should follow these lines [14].

Research studies performed on animal models reported an incremental mean area per unit of collagen fibers in their posterior limb compared with the abdominal area. That is because the limb is exposed to continuous tensions during movements compared to other parts of animals [15].

Changes in the pattern of arrangement of collagen fibers were also observed in the animal models with the inference of direction of extracellular matrix changes according to the type of stress and their movements [16, 17]. This supports the anatomical arrangement of the collagen fibers that lie more or less parallel to the epidermis in abdomen area when compared to perpendicular arrangement to the epidermis in posterior limb of animals [15].

These results probably uphold the fact that scar behavior will be different at different age due to the difference in production of collagen and elastic proteins in the human body as the age advances. In addition to age factor, chronic exposure of the skin to UV rays [18] or treatment with laser light [19] also affect the collagen content of scar. Therefore, the wounds would heal more slowly and the skin will be more vulnerable to environmental stressors [2].

In conclusion, the significant differences in correlations among the collagen and elastic fiber content of the skin in different areas of extremities are dependent on the effect of localized forces. Hence, the linear association of dermal collagen and elastic fibers in the different region and different directions, apart from tension lines, could be one of the possible anatomical reasons of unpredictable scar behavior in different areas and different orientations of the same area.

## Figures and Tables

**Table 1:** Spearman's correlation coefficient (*r*) and its level of significance (*p*) for pattern at extremities.

Variables between	Shoulder joint		Wrist		Ankle		Forearm		Thigh	
	*r*	*p*	*r*	*p*	*r*	*p*	*R*	*p*	*r*	*p*
C_H_ & C_V_	0.66	**0.000** ^**#**^	0.75	**0.000** ^**#**^	0.26	**0.14**	0.75	**0.000** ^**#**^	0.80	**0.000** ^**#**^
C_H_ & E_H_	−0.15	**0.39**	0.01	**0.9**	0.11	**0.54**	−0.14	**0.41**	−0.65	**0.000** ^**#**^
C_V_ & E_V_	−0.30	**0.94**	−0.01	**0.92**	0.23	**0.20**	−0.51	**0.003** ^**#**^	−0.25	**0.15**
E_H_ & E_V_	−0.34	**0.05**	0.20	**0.26**	0.19	**0.28**	0.65	**0.000** ^**#**^	0.42	**0.01** ^**∗**^

^#^ *Correlation is significant at 0.01 level *(*p* < 0.01). ^*∗*^*Correlation is significant at 0.05 level *(*p* < 0.05) (C_H_: collagen in horizontal direction; C_V_: collagen in vertical direction; E_H_: elastic in horizontal direction; E_V_: elastic in vertical direction).

**Table 2:** Comprehensive correlation pattern of dermal collagen and elastic fibers between horizontal and vertical directions at extremities.

	Between C_H_ and C_V_	Between C_H_ and E_H_	Between C_V_ and E_V_	Between E_H_ and E_V_
Shoulder joint	Moderate positive (*r* = 0.66)	-	-	-
Wrist	Strong positive (*r* = 0.75)	-	-	-
Ankle	-	-	-	-
Forearm	Strong positive (*r* = 0.75)	-	Moderate negative (*r* = −0.51)	Moderate positive (*r* = 0.65)
Thigh	Strong positive (*r* = 0.80)	Moderate negative (*r* = −0.65)	-	Low positive (*r* = 0.42)

*r: Spearman's correlation coefficient *(C_H_: collagen in horizontal direction; C_V_: collagen in vertical direction; E_H_: elastic fibers in horizontal direction; E_V_: elastic fibers in vertical direction).

**Table 3:** Summary of dominance pattern of dermal collagen and elastic fiber content at extremity region. C_H_: Collagen in horizontal direction; C_V_: collagen in vertical direction; E_H_: elastic in horizontal direction; E_V_: elastic in vertical direction.

	C_H_	C_V_	E_H_	E_V_
Shoulder joint	↑			↑
Wrist		↑		↑
Thigh	-	-	↑	
Ankle	-	-		↑
Forearm	-	-	-	-

↑:* significantly increased content between horizontal and vertical directions.*

## Additional Points

*Limitations of the Study*. In the present study, we have analyzed the data of samples obtained from the male human cadavers with the age ranging from 55 to 70 years. Due to nonavailability of female cadavers and the other age ranges, the comparison of the effect of age and sex on the correlation coefficient could not be performed.
